# Porcine Models of Neurotrauma and Neurological Disorders

**DOI:** 10.3390/biomedicines12010245

**Published:** 2024-01-22

**Authors:** John C. O’Donnell, Dmitriy Petrov

**Affiliations:** 1 Center for Neurotrauma, Neurodegeneration & Restoration, Corporal Michael J. Crescenz Veterans Affairs Medical Center, Philadelphia, PA 19104, USA; 2 Center for Brain Injury & Repair, Department of Neurosurgery, Perelman School of Medicine, University of Pennsylvania, Philadelphia, PA 19104, USA

The translation of therapeutics from lab to clinic has a dismal record in the fields of neurotrauma and neurological disorders. This is due in part to the challenging heterogeneity of the clinical population common to all translational research, but also due to the unique challenges of recreating the mechanisms and manifestations of human neurological injury/disorders in small animals. Large animal models are an essential component of successful pipelines for moving discoveries from bench to bedside in other fields when exploring device or therapeutic scale-up and/or investigational new drug/investigational device exemption (IND/IDE)-enabling studies, and neuroscience has made significant progress toward establishing such pipelines in its many unique subfields. Due to their size, neuroanatomy, and other factors, pigs have proven to be ideal for providing high-fidelity, clinically relevant studies to bridge the gap between small animals and humans.

This Special Issue collects a dozen papers from leaders in the fields of neurotrauma and neurological disorders detailing clinically relevant studies and sophisticated swine model systems that demonstrate their potential to empower translational research. There are five primary research papers utilizing the rotational acceleration injury model of traumatic brain injury (TBI), which requires a brain of sufficient mass (like the pig) to generate injurious forces. Dr. Grovola and colleagues in the Cullen laboratory reported hyper-ramified microglia in the gray matter after mild TBI, with fibrinogen indicative of blood–brain barrier disruption also predominantly in the gray matter, adding to the body of evidence that rotational acceleration pathology is not limited to white matter [1]. The Margulies group contributed three primary research articles utilizing rotational acceleration in a pediatric pig model. Oeur et al. applied single and repeated low-velocity head rotations in piglets and also gathered data from adolescent humans presenting with concussion, and found that deficits in the pupillary light reflex were altered after injury compared to reference ranges, suggesting that pupillometry could be a valuable tool for neurofunctional assessment [2]. Mull et al. reported significant gait alterations in piglets following rotational acceleration that were more severe and longer lasting when multiple rotations were applied, and they also validated reference ranges for assessing gait alterations in piglets [3]. Dr. Oeur and colleagues also investigated auditory and visually evoked potentials following rotational acceleration in piglets, and found that single and repeated injury groups exhibited different alterations to their evoked potential responses [4]. Finally, we reported the development of neurocritical care techniques and a neurointensive care unit stocked with clinical equipment for use in pigs, which allowed us to extend the study period for TBI with coma in pigs beyond 8 h for the first time while gathering extensive, clinically relevant neuromonitoring data [5].

Beyond the papers featuring rotational acceleration injury, Dr. Shin and colleagues in the Kilbaugh laboratory reported on the efficacy of a promising biological approach targeting phosphorylated tau and reducing pathology after a controlled cortical impact (CCI) in pigs [6]. Additionally, Pavlichenko and Lafrenaye employed central fluid percussion injury (CFPI) in pigs and found that while cellular signs of pathology and inflammation correlated with the pressure pulse, blood gasses and mean arterial pressure did not; furthermore, they reported that preserved, refrigerated pig brain tissue was histologically viable after 10 years [7]. We also received papers detailing new tools for use in pig neurotrauma studies. Knibbe and colleagues from the Boakye laboratory detailed the use of the SmartPill™ device for data collection from the gastrointestinal tract following spinal cord injury [8]. Ao and colleagues from the Vonder Haar laboratory also described a new reinforced touchscreen system for administering behavioral assessments in pigs [9]. This behavioral touchscreen work ties into a review in this collection from Alesa Hughson Netzley that provides a wider overview of behavioral testing techniques for use in pig models of neurotrauma, following up on their previous behavioral work in Yucatan minipigs [10,11]. We also provided a review, led by Erin Purvis, of astrocytic studies in pigs, including tables of antibodies that were utilized in various studies, as we are sympathetic to the difficulties associated with finding antibodies and reagents compatible with pigs [12]. Finally, Dr. Wathen and colleagues provided an extensive review of spinal cord injury studies in pig models and the significant translational advantages that pigs present [13].

Recognizing the advantages (e.g., translational, logistical) as well as the limitations of the models that we utilize is vital for accurate reporting and establishing implications of clinical relevance. Such honest assessment of preclinical models is necessary for a functional translational pipeline, and worthy of a brief discussion in this editorial. As the majority of the papers in this collection came from TBI research, we will examine preclinical TBI modeling as an example.

**Recognizing the Strengths and Limitations of Preclinical TBI Models**

Over the past several decades, the neurotrauma field has wrestled with a lamentable translational failure rate. In particular, although hundreds of treatments have shown efficacy in rodent models of TBI, none have shown effectiveness in humans, despite over 30 clinical trials strongly supported by preclinical rodent data [14,15,16,17]. The failure of these trials has led to several National Institute of Neurological Disorders and Stroke (NINDS) and Department of Defense (DoD) conferences which have concluded that validated biomarkers of underlying pathological mechanisms are critical for (1) the proper classification of this heterogeneous disease and (2) the development of effective therapies that target specific injury mechanisms [18,19]. Although challenges in clinical trial design and the heterogeneity of human TBI contribute to the lack of positive findings in clinical studies, this dismal translational record also highlights the limitations of rodent models that simply cannot replicate many of the core mechanisms and manifestations of human TBI.

The vast majority of clinical TBIs are closed-head diffuse brain injuries caused by a blow or rapid jolt to the head, causing abrupt motion of the brain within the skull. In moderate-to-severe cases, the acute post-injury phase is marked by rapid cell death and axonal disruption, generally occurring as a direct consequence of biomechanical loading that surpasses cellular/axonal thresholds. In closed-head TBI in humans, the severity of the biomechanical loading is a product of brain mass, neuroanatomy, and head rotational acceleration [20,21,22]. Therefore, animal models that feature a species with a large brain mass, a gyrencephalic brain architecture, and head rotational acceleration as the inertial loading mechanism are uniquely able to apply biomechanical parameters scaled from those known to occur—and be injurious—in human TBI [20,21,22,23]. Indeed, pigs possess a large brain mass and gyrencephalic architecture with a 60:40 white/gray matter ratio as found in the human brain; in contrast, rats and mice present a paucity of white matter, resulting in 14:86 and 10:90 white/gray matter ratios, respectively [24,25,26]. This is particularly important because diffuse axonal injury (DAI)—predominantly a white matter affliction—has been described as the “hallmark” pathology of closed-head diffuse TBI. White matter in the brain consists of long viscoelastic axon tracts that can stretch to accommodate slow shearing forces, but intra-axonal components may snap when exposed to the shear deformation forces generated by rapid acceleration [27,28]. Moreover, the most commonly employed rodent models of TBI rely on either a focal impact to the brain surface (controlled cortical impact—CCI) or a sharp increase in intracranial pressure surrounding the brain (fluid percussion injury—FPI); however, these loading mechanisms do not reproduce the mechanical forces of inertial TBI in humans, nor do the injuries produce the same clinical manifestations observed after human TBI. For example, when compared to the clinical TBI severity criteria from which they derive their classification terminology, rodent models of “mild” TBI typically result in cortical lesions that do not align with the criteria of a closed skull and an absence of lesions in imaging necessary for clinical classification as mild TBI, and even the most “severe” TBI injuries in rodents do not produce loss of consciousness sufficient even for clinical classification as moderate TBI, let alone severe. The misapplication of these terms to rodent models in the preclinical TBI literature is a major source of confusion, and confusion negatively impacts progress in any field of research. While moderate or severe human TBI may involve pathology from a combination of impact-loaded focal injury and rotational acceleration-loaded diffuse injury, the most obvious manifestation of a moderate-to-severe TBI—protracted loss-of-consciousness >60 min—requires damage from rotational acceleration in closed-head TBI, and cannot be produced via linear acceleration or impact alone [29,30,31,32].

Injury via head acceleration has been attempted in rats, but while bleeding was observed, the conclusions were revealed to be flawed in an expert commentary noting that no consideration was given to scaling up acceleration to account for the much smaller brain mass of rats [33,34]. These biomechanical scaling parameters have been extensively studied, and to generate the injurious forces of human TBI in a rat, head acceleration would need to be increased by an unfeasible 8000% relative to human head acceleration [22,34,35]. Despite the commentary associated with the original article that clearly established the fatal flaws of the study and the physical impossibility of administering injury via acceleration in the rodent brain, the original paper was not retracted, and as a result, head acceleration was subsequently revisited in rodent models 14 years later via the deceptively-named CHIMERA (Closed-Head Impact Model of Engineered Rotational Acceleration) system that allows for head movement after impact. Despite the continued spread of misinformation via the CHIMERA model, the fact remains that rodent brain mass is far too small to reach scaled thresholds for generating injurious forces from rotational acceleration, and the pathology—while presenting with a diffuse gradient—emanates from the impact site, as seen in other impact loading models [36,37]. The injurious forces generated by acceleration are highly dependent on brain mass, and so to replicate forces present in a larger human brain during TBI, the acceleration of smaller brains must be scaled up accordingly. Impact loading does not scale with brain mass, so administering an impact powerful enough to scale up the resultant head acceleration would lead to a large discrepancy in the proportion of impact-to-acceleration loading when compared to human TBI. Therefore, even in large gyrencephalic animals like swine with brains closer in size to humans, an unscaled impact and scaled acceleration must be administered separately to replicate the mechanisms of human TBI. Overall, the fundamentally flawed CHIMERA model has created a great deal of confusion in our field, and the scientific record is in dire need of correction. Fortunately, after removing all inaccurate references to and claims of acceleration-loaded injury in small animals, researchers may be left with a valid—if somewhat overcomplicated—CCI model, and therefore many authors duped into utilizing CHIMERA may be able to salvage their publications via errata rather than retraction.

While small animal models cannot reproduce the same biomechanical perturbations as human TBI, they clearly produce a mechanical injury to the rodent brain, and while the clinical manifestations of TBI do not line up with the manifestations of the small animal model injuries, many of the individual endophenotypes of human TBI can be reproduced in some fashion. Therefore, small animal models remain an essential tool for studying the secondary injury mechanisms of TBI, identifying potential therapeutic targets and candidates, and studying the mechanism of action of novel therapeutics. These essential basic and early-phase translational studies require the accessibility and affordability of small animal models—enabling high-throughput testing—while the recognition of limitations and measured interpretation are necessary to improve applicability to clinical TBI. However, a more effective translational pipeline for developing TBI therapeutics should feature pre-IND/IDE studies in large gyrencephalic animal models that incorporate inertial loading with angular rotational acceleration—to better recapitulate the biomechanical causation and pathophysiology of the majority of human TBIs—prior to translation into clinical trials. This will better inform clinical trials (e.g., inclusion criteria, key outcomes, etc.) and allow novel therapeutic candidates to fail early when tested in the context of the mechanisms and manifestations of human injury, thereby avoiding the expensive failures in clinical trials that have stymied industry investment in neurotrauma. The rotational acceleration model is currently only in use at the University of Pennsylvania and Georgia Tech./Emory University, creating a considerable bottleneck for the translation of therapeutics. It will take a concerted effort from funding agencies and research universities to establish additional sites that can utilize this model. Establishing additional sites of rotational acceleration TBI research (which requires extensive training with large gyrencephalic animals and a powerful pneumatic actuator) is a daunting but necessary task if we want a viable translational pipeline for TBI. While TBI is a special case, many of these lessons are applicable to the preclinical study of stroke, spinal cord injury, and other facets of neurotrauma and neurological disorders. Only by recognizing the strengths and limitations of our models can we hope to effectively develop, validate, and translate novel neuroprotective and regenerative therapeutics into clinical practice to maximize functional recovery.
